# Pseudo-postpacing interval of diastolic potential after entrainment pacing of remote bystander pathway in reentrant ventricular tachycardia

**DOI:** 10.1007/s00380-013-0411-5

**Published:** 2013-09-24

**Authors:** Yoshiaki Kaneko, Tadashi Nakajima, Tadanobu Irie, Osamu Igawa, Takafumi Iijima, Masaki Ota, Mio Tamura, Takashi Iizuka, Shuntaro Tamura, Akihiro Saito, Masahiko Kurabayashi

**Affiliations:** 1 Department of Medicine and Biological Science, Gunma University Graduate School of Medicine, 3-39-22 Showa-machi, Maebashi, Gunma 371-8511 Japan; 2 Department of Internal Medicine and Cardiology, Nippon Medical School Tama-Nagayama Hospital, Tokyo, Japan

**Keywords:** Ventricular tachycardia, Reentrant tachycardia, Entrainment mapping, Postpacing interval, Critical isthmus, Remote bystander pathway

## Abstract

After entrainment pacing, the postpacing interval of a diastolic potential may be misinterpreted if the distal tip of the ablation catheter captures a remote bystander pathway adjacent to the critical isthmus of a complex reentrant circuit in a structurally diseased heart. We discuss this possible pitfall of entrainment mapping of reentrant ventricular tachycardia, observed after a healed myocardial infarction.

## Introduction

Entrainment mapping is a key maneuver used to locate the critical isthmus of a reentrant circuit during catheter ablation of a reentrant ventricular tachycardia (VT). [1] The postpacing interval (PPI) of the diastolic potential (DP), reflective of the activation in the zone of slow conduction following entrainment pacing limited to the critical isthmus, is typically equal to the tachycardia cycle length (TCL) [1], whereas the PPI of a DP during entrainment pacing outside the critical isthmus is of electrophysiologic interest [2, 3]. We describe the electrophysiologic behavior and significance of the PPI after entrainment pacing of a remote bystander pathway near the reentry circuit of VT after a healed anteroseptal myocardial infarction, followed by autopsy of the heart.

## Case report

**Fig. 1:**
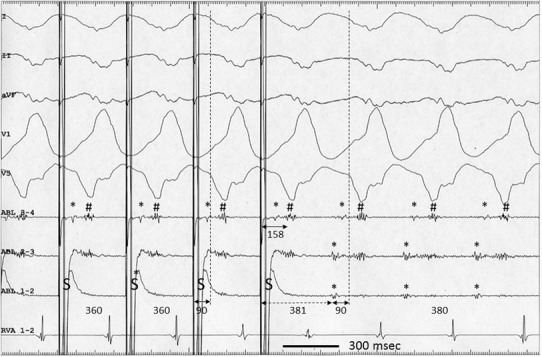
Surface electrocardiograms and intracardiac recordings during entrainment pacing from electrode 1–2 of the ablation catheter (ABL), at a cycle length of 360 ms. The QRS morphology during pacing was similar to that during VT, consistent with concealed entrainment. The 90-ms interval (*left bidirectional solid arrow*) between pacing stimuli (*S*) and onset of QRS (*left vertical dashed line*) is equal to the 90-ms interval (*right solid bidirectional arrow*) between diastolic potential (DP) (*asterisk*) and onset of QRS (*right vertical dashed line*). The 381-ms postpacing interval (PPI) of the DP (*dashed bidirectional arrow*) is nearly equal to the 380-ms tachycardia cycle length (TCL). The interval between S and the ventricular electrogram (*hash*) recorded from ABL 3–4 was 158 ms. See text for further explanations. *I, II, V1* surface electrocardiogram, *ABL 3–4 to 1–2* proximal and distal bipoles of the ABL, *RVA* right ventricular apex

**Fig. 2:**
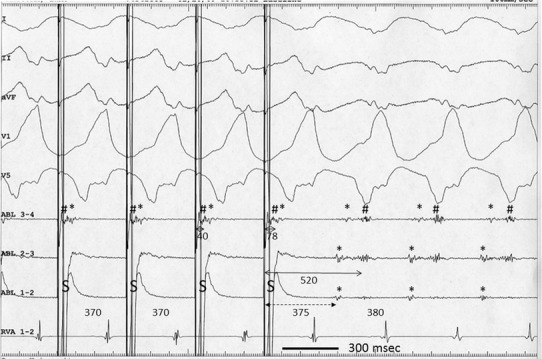
Surface electrocardiograms and intracardiac recordings during entrainment pacing from electrode 1–2 of the ablation catheter (ABL) at a cycle length of 370 ms. During pacing, the QRS complexes are fused, with absence of an S-QRS delay. The 40-ms interval between the pacing stimulus (*S*) and the ventricular electrogram (*hash*), recorded from ABL 3–4, is shorter than in Fig. 1. The 78-ms interval between S and DP (*asterisk*) recorded from ABL 3–4 is the same as in Fig. 1. The 520-ms PPI of the local ventricular electrogram (true PPI) (*bidirectional thin arrow*) is longer than the 380-ms TCL, whereas the 375-ms PPI of the DP (false PPI) (*bidirectional dashed arrow*) is shorter than the 380-ms TCL. A 40-ms interval is recorded from ABL 3–4 between S and the ventricular electrogram. See text for further explanations. Abbreviations as in Fig. 1

**Fig. 3:**
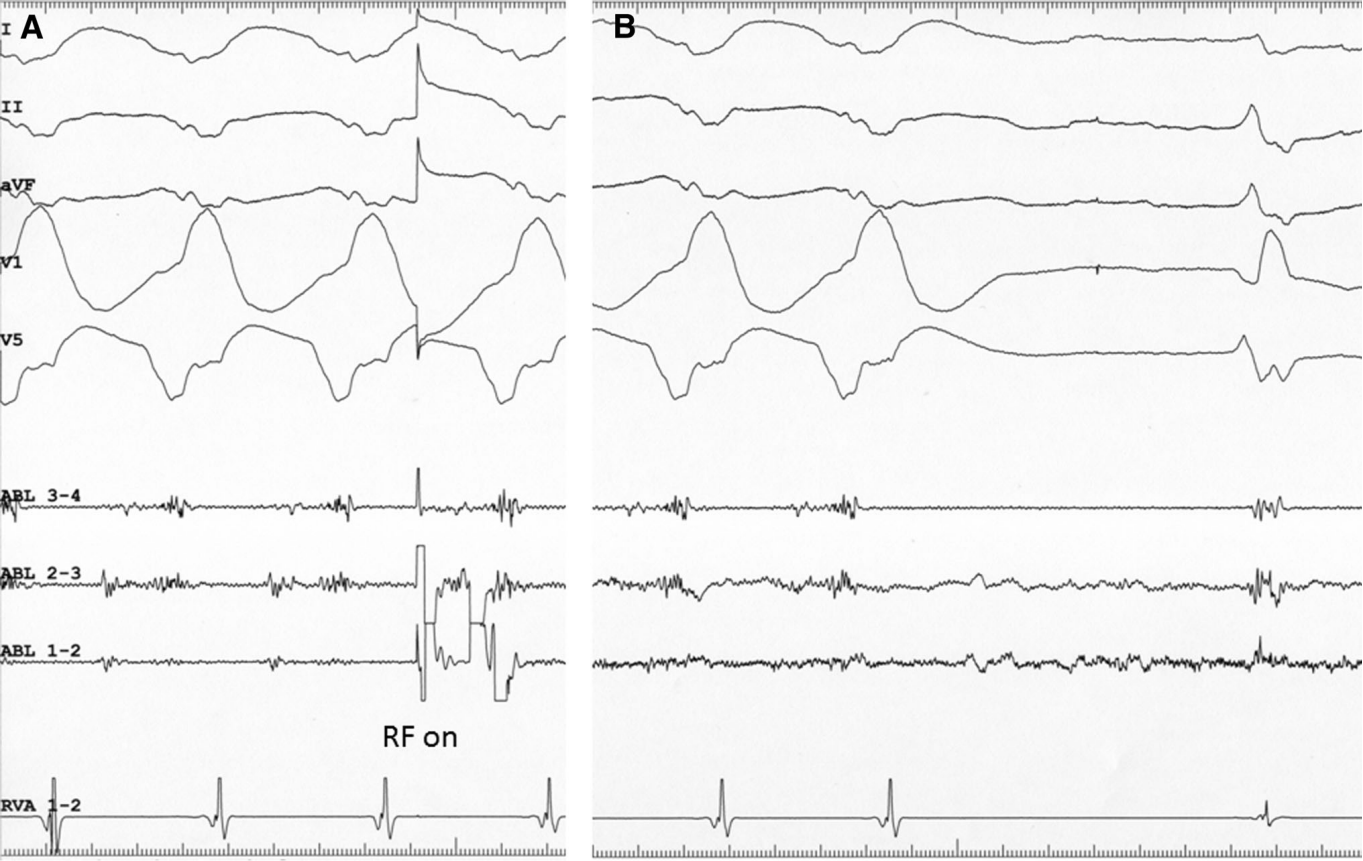
**a** Intracardiac electrograms at the site of successful ablation immediately before radiofrequency (RF) delivery (*RF on*). **b** Intracardiac electrograms recorded during RF delivery, at the time of termination of the tachycardia. neither the DP nor the local ventricular electrogram recorded during the application of RF at the site of successful ablation decreased significantly. Abbreviations as in Fig. 1

**Fig. 4:**
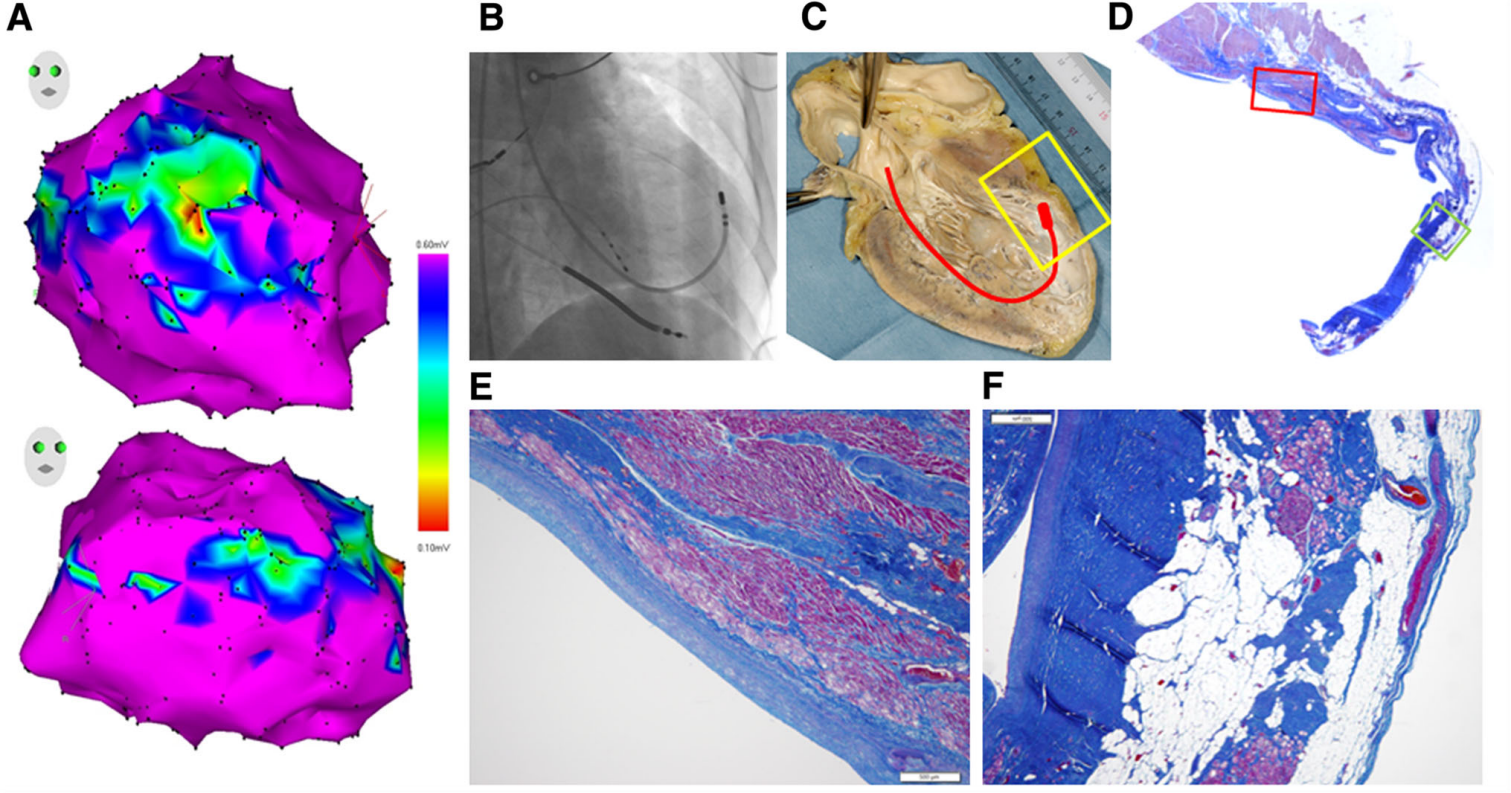
**a** Electroanatomic map of the left ventricle, including voltage map during sinus rhythm. Healthy tissue >0.6 mV is represented by the color *purple*, scar border (0.1–0.6 mV), *blue/green/yellow*, and scar (<0.1 mV), *red*. **b** Right oblique fluoroscopic view of the ablation catheter (ABL) with its tip located at the site of successful ablation. **c** Macroscopic section of the left ventricle along the plane shown in **a**, with schematic representation of the ablation catheter with its tip at the basal margin of the aneurysm. **d** Low-power light-microscopic view of the zone of anterior aneurysm corresponding to the *yellow rectangle* shown in **b**; the *red rectangles* show the higher-power microscopic areas shown at the base (*red rectangle*) and in the apical region of the aneurysm (*green rectangle*). **e** High-power microscopic section at the basal margin of the aneurysm; **f** High-power microscopic section of the apical aneurysmal region (color figure online)

A 73-year-old man with a history of healed anteroseptal myocardial infarction underwent radiofrequency (RF) catheter ablation of VT with right bundle branch block and undetermined QRS axis morphology, refractory to drug therapy. The patient had undergone implantation of a cardioverter-defibrillator 2 years earlier. A left ventriculography revealed the presence of anterior wall dyskinesia and a 20 % left ventricular ejection fraction. An area of low-amplitude bipolar electrograms, defined as ≤ 0.6 mV [4], was present on a CARTO-X-P electroanatomic map (Biosense Webster, Diamond Bar, CA, USA) of the anterior left ventricular endocardial surface, recorded during sinus rhythm (Fig. 4a). VT was reproducibly induced by programmed ventricular stimulation from the right ventricular apex. Pacing during VT at the basal edge of the low-voltage zone, where a low-amplitude DP was recorded, showed concealed entrainment, with a pacing stimulus-QRS equal to the DP-QRS interval, and a PPI of the DP equal to the TCL, indicating that the pacing site was located on the critical isthmus of the reentrant circuit (Figs. 1, 4b). Further entrainment from the same site, at a slightly longer paced cycle length and identical pacing output, caused constant fusion without pacing stimulus-QRS delay, and a PPI of the DP nearly similar to the TCL (Figs. 2, 4b). The delivery of RF energy for 21 s at that site, using a 4-mm-tip, nonirrigated catheter at a maximum power of 50 W and maximum temperature of 60 °C, terminated and eliminated the inducibility of VT (Fig. 3) [5, 6].

The patient died of incessant VT 1 year after the ablation procedure, and a postmortem examination of the heart was performed. The heart weighed 590 g. The left ventricle was open across the anterior aneurysm and the apex (Fig. 4c), along the same plane as the right oblique fluoroscopic view shown in Fig. 4b, exposing the ablation region. On visual inspection the left ventricle was not dilated, although the posterior and septal walls were hypertrophied, and a 6 cm × 5 cm × 2 mm anterior aneurysm was present, with a trabeculated and whitish endocardial surface (Fig. 4c). The site of successful ablation was at the basal margin of the aneurysm (Fig. 4b, c). The aneurysm was sectioned transmurally, perpendicular to the mitral annulus, stained with Masson trichrome, and processed for standard microscopic examination (Fig. 4d), which revealed nontransmural fibrosis within two-thirds of the aneurysmal endomyocardium, corresponding to the ablation lesion (Fig. 4e), and nonablated, surviving endomyocardium adjacent to the aneurysm (Fig. 4f).

## Discussion

**Fig. 5:**
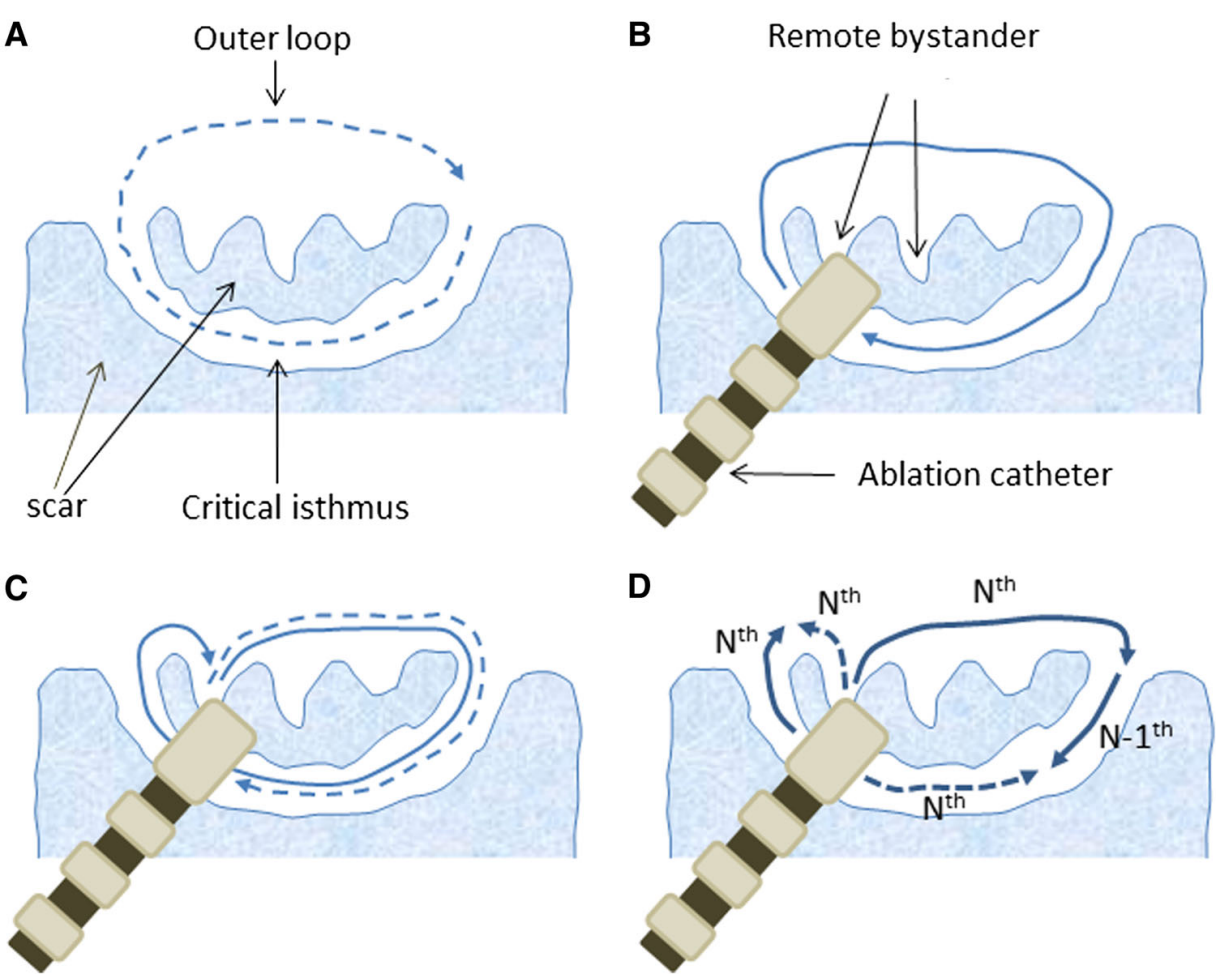
Schematic representation of the reentry circuit (**a**), tentative explanations of the PPI after the last capture in Figs. 1 and 2 (**b** and **c**, respectively), and the impulse propagation during entrainment pacing in Fig. 2 (**d**). The critical isthmus across the scar and outer loop outside the scar are represented. The tip of the multipolar ablation catheter positioned on the critical isthmus, adjacent to the remote bystander, is illustrated in **b** and **c**. The *dashed arrow* in **a** shows the direction of activation in the reentry circuit, corresponding to the wavefront propagation during tachycardia. The *solid arrows* in **b** and **c**, and the *dashed arrow* in **c** show the direction of activation in the reentry circuit, corresponding to the wavefront of the true and pseudo-PPI, respectively. The *solid* and *dashed arrows* in **d** show the respective propagations of the orthodromic and antidromic wavefronts. See text for further explanations

The morphology, amplitude, and timing of the local ventricular electrograms and DP relative to the QRS complex recorded from the ablation catheter during VT are similar in Figs. 1 and 2, confirming that the tip of the ablation catheter was in the same position relative to the critical isthmus during both episodes of entrainment pacing. Since, as shown in Figs. 1 and 2, the 90-ms DP-QRS interval represents <30 % of the 380-ms TCL, the pacing site was located near the exit of the critical isthmus (Fig. 5b) [1]. However, in contrast to Fig. 1, no latency of the pacing stimulus-QRS complex associated with a short, 40-ms interval between the pacing stimulus and the local ventricular electrogram is visible during entrainment pacing in Fig. 2, suggesting that, unlike the capture limited to the critical isthmus in Fig. 1, pacing in Fig. 2 captured the ventricular myocardium outside the zone of slow conduction. When entrainment pacing captured the ventricular myocardium outside the zone of slow conduction, the orthodromic wavefront reached the recording site of the DP in the critical isthmus without colliding with the antidromic wavefront (Fig. 5c). Therefore, in contrast to the PPI of the DP shown in Fig. 1, that shown in Fig. 2 is not a true PPI, generally defined as the interval between the last pacing stimulus and the subsequent, directly captured electrogram [1]. The true PPI in Fig. 2 corresponded to the interval between the last pacing stimulus and the captured ventricular electrogram, obviously longer than the TCL, suggesting that pacing in Fig. 2 captured a remote bystander pathway (Fig. 5c) [7]. Consequently, the PPI of the DP in Fig. 2 corresponds to the interval between the pacing site in the remote bystander pathway and the DP recorded in the critical isthmus (pseudo-PPI) (Fig. 5c). Furthermore, that the PPI of the DP is nearly equal to the TCL in Fig. 2 does not mean that the pacing site was on the critical isthmus. Although the interval between the pacing stimulus and the DP recorded at the proximal electrode of the ablation catheter during entrainment pacing was similar in Figs. 1 and 2, it is not clear that pacing in Fig. 2 also captured the critical isthmus. Even in the presence of simultaneous capture of the essential and remote bystander pathways, neither the orthodromic nor the antidromic wavefronts originating from pacing at the critical isthmus would affect the orthodromic wavefront created by pacing at the remote bystander pathway, for the following reasons. When entrainment pacing captures the critical isthmus and the remote bystander simultaneously (as illustrated in Fig. 5d), the *N*th antidromic wavefront originating from pacing at the remote bystander collides constantly, probably outside the critical isthmus, with the *N*th orthodromic wavefront created by pacing at the critical isthmus, representing atypical constant fusion (Fig. 5d). Furthermore, the *N*th antidromic wavefront originating from pacing at the critical isthmus collided within the critical isthmus, with the *N − 1*th orthodromic wavefront created by pacing at the remote bystander (Fig. 5d). Finally, the *N*th orthodromic wavefront created by pacing at the remote bystander pathway reached the recording site of the DP in the critical isthmus, without colliding with the antidromic wavefront (Fig. 5c). Consequently, the PPI corresponds to the interval between the pacing site in the remote bystander and the DP recording in the critical isthmus (Fig. 5c).

This was probably due to simultaneous contact of the distal tip of the ablation catheter with the critical isthmus and the neighboring remote bystander pathway, along with a slight change in the contact with the endocardium associated with a minimal change in the paced cycle length, without a change in the pacing output/pulse width of 5 V/0.5 ms. The pathologic-fluoroscopic correlation suggested that the critical isthmus and the bystander pathway were consistent with the ablated, basal margin of an aneurysm and surviving endomyocardium adjacent to the aneurysm, respectively, both of which could be simultaneously in contact with the tip of the ablation catheter (Fig. 4).

In summary, entrainment pacing capturing the remote bystander pathway simultaneously with a recording of the DP represented a pseudo-PPI of the DP. This phenomenon, which may be observed in complex reentrant circuits with a critical isthmus located near the remote bystander, is not rare. The pseudo-PPI of the DP can be misinterpreted as the true PPI, especially when constant fusion causing minimal QRS fusion and simultaneous recording of the DP is misidentified as concealed entrainment. When entrainment pacing captures the remote bystander pathway simultaneously with a recording of a DP, causing constant fusion, the PPI of the DP does not represent the true PPI. In some circumstances, the pseudo-PPI might be shorter than the tachycardia cycle length [2, 3]. The absence of QRS fusion, i.e., concealed entrainment, must be confirmed during entrainment pacing to measure the true PPI of the DP.
